# Feasibility of Laparoscopic Nephrectomy in Children With Hydronephrotic Kidneys: A Prospective Study

**DOI:** 10.7759/cureus.95190

**Published:** 2025-10-22

**Authors:** Shariq Anis Khan, Muhammad Ahsan Rehman Khan, Fouzia Naeem Effendi, Osama Kalim Shaikh, Salman El Khalid, Muhammad Idrees

**Affiliations:** 1 Urology, The Kidney Centre Postgraduate Training Institute, Karachi, PAK; 2 Community Health Sciences, Bahria University Health Sciences Campus, Karachi, PAK; 3 Urology, Dow University of Health Sciences - Ojha Campus, Karachi, PAK

**Keywords:** hydronephrosis, laparoscopic nephrectomy, pediatric urology, surgical outcomes, transperitoneal approach

## Abstract

Background

Laparoscopic nephrectomy has become gradually popular in pediatric cases related to urology because of its minimally invasive nature and favorable outcomes. However, its feasibility in hydronephrotic, non-functioning kidneys remains a technical challenge in children due to anatomical distortion and potential adhesions.

Objective

The main objective of this study is to assess the feasibility, safety, and outcomes of transperitoneal laparoscopic nephrectomy in children with hydronephrotic kidneys.

Methods

The prospective observational research was carried out at The Kidney Centre Postgraduate Training Institute, Karachi, Pakistan, from May 2019 to August 2023. A total of 102 pediatric patients with non-functioning hydronephrotic kidneys underwent laparoscopic nephrectomy. Demographic, intraoperative, and postoperative parameters were recorded. The statistical study included descriptive statistics and the Chi-square test for associations.

Results

The median age was seven years, with 62 males (60.8%) and 40 females (39.2%). The left kidney was more commonly affected (57, 55.9%). Pelviureteric junction obstruction (PUJO) was the most frequent etiology (46, 45.1%), followed by stone disease (39, 38.2%) and vesicoureteral reflux (VUR) (17, 16.7%). Laparoscopic nephrectomy was completed in 96 patients (94.1%), with six (5.9%) conversions to open surgery. The mean operative time was 93.5 ± 18.7 minutes, and the mean blood loss was 72.4 ± 25.6 mL. Intraoperative complications occurred in five patients (4.9%), and postoperative complications in seven (6.9%) - all low-grade. Conversion was significantly associated with PUJO (p = 0.034), longer operative duration (p = 0.001), and more blood loss (p = 0.004). No significant associations were found with gender or kidney side.

Conclusion

The findings of the research show that transperitoneal laparoscopic nephrectomy is a feasible and safe option in children with hydronephrotic kidneys, demonstrating low complication and conversion rates when performed by experienced surgeons.

## Introduction

Nephrectomy is among the most frequently performed ablative procedures in urology. It is utilized to manage a variety of benign, as well as malignant, cases affecting the kidney and upper urinary tract [1]. Since Gustav Simon performed the first nephrectomy in 1869, the procedure has evolved significantly [2]. In recent decades, minimally invasive techniques have increasingly replaced open nephrectomy, especially in the pediatric population, due to their many advantages [3].

Laparoscopic nephrectomy gained widespread acceptance after Clayman's landmark description in adults in 1990, and Ehrlich’s report of the first pediatric case in 1992 [4]. Compared to open surgery, the laparoscopic approach offers fewer incisions, reduced postoperative pain, less blood loss, reduced hospital stays, quicker recovery, and improved cosmetic outcomes [5,6]. In children, the most commonly indicated factors for nephrectomy include non-functioning kidneys caused by pelviureteric junction obstruction (PUJO), vesicoureteral reflux (VUR), ectopic ureters, or multicystic dysplastic kidney (MCDK) [4].

Two primary approaches are used in laparoscopic nephrectomy: transperitoneal and retroperitoneal. The transperitoneal route offers a wider operative field and better visualization of adjacent organs, while the retroperitoneal method, although technically more demanding because of limited working area, can allow faster recovery by sparing intra-abdominal organs [7].

In Pakistan, limited data exist on pediatric laparoscopic nephrectomy, particularly in children with hydronephrotic kidneys. Hydronephrosis presents additional technical challenges during laparoscopic nephrectomy due to the distended collecting system, which restricts working space and complicates dissection. These difficulties may be further intensified by adhesions resulting from infections or stone-related inflammation [4].

The development of pediatric laparoscopy began with Cortesi et al.'s use of diagnostic laparoscopy in 1976 to locate non-palpable undescended testes [8]. Since then, it has expanded to include pyeloplasty, partial nephrectomy, and nephrectomy [9]. However, its adoption has been relatively slower in children due to the proven efficacy of open surgery, the small size of the pediatric abdomen, and the limited availability of appropriately sized instruments [10].

While conventional nephrectomy, performed through large flank or subcostal incisions, remains effective, laparoscopic nephrectomy has gained favor due to its shorter hospital stay, superior cosmetic outcomes, and manageable learning curve [11]. Both the transperitoneal and retroperitoneal approaches have shown comparable clinical outcomes, with the choice of technique largely depending on surgeon preference and expertise [12].

Given the added technical complexities associated with hydronephrotic kidneys in children, it is essential to evaluate whether laparoscopic nephrectomy remains a safer and more effective approach. This study was therefore undertaken to address the lack of local data and evaluate the feasibility and outcomes of transperitoneal laparoscopic nephrectomy in children with hydronephrotic kidneys.

## Materials and methods

This prospective observational study was conducted over a period of four years and four months, from May 2019 to August 2023, at The Kidney Centre Postgraduate Training Institute, Karachi, Pakistan, after taking ethical approval (Reference number: 161-URO-192019; dated May 19, 2019). All procedures were executed single-handedly by an experienced pediatric urologist, ensuring uniformity in surgical approach, perioperative decision-making, and data collection.

The sample size was calculated using OpenEpi version 3.01, considering a confidence level of 95%, an error margin of 5%, and a probable feasibility rate of 50% (due to the absence of large-scale regional data). This produced a required sample size of 102 patients, which was achieved during the study duration.

The study employed a non-probability consecutive method of sampling. All pediatric patients visiting the Urology Department during the study period with non-functioning hydronephrotic kidneys who met the eligibility criteria were included consecutively until the required sample size was reached.

Inclusion criteria consisted of patients aged 6 months to 14 years, who were diagnosed with unilateral non-functioning hydronephrotic kidneys based on radionuclide renal scans (diethylenetriamine pentaacetic acid (DTPA) or mercaptoacetyltriglycine (MAG3)) showing <10% differential renal function. All patients were evaluated preoperatively with abdominal ultrasonography, renal scintigraphy, and contrast-enhanced CT urograms, where necessary. Only those patients who were planned for laparoscopic nephrectomy as a primary treatment modality were included.

Exclusion criteria included patients with bilateral renal disease, anatomical abnormalities precluding laparoscopic access, coagulopathy, active urinary tract infections, or significant cardiopulmonary comorbidities contraindicating general anesthesia. Patients who had a history of any major abdominal surgery were also excluded due to the increased risk of adhesions interfering with laparoscopic access.

All patients underwent a standardized preoperative evaluation, including complete blood count, renal function tests, coagulation profile, urinalysis, abdominal ultrasound, and DTPA/MAG3 scans. Contrast-enhanced CT urogram was performed selectively in cases with unclear anatomy on ultrasound or radionuclide scan.

All surgeries were carried out under general anesthesia, along with endotracheal cannulation. Laparoscopic transperitoneal nephrectomy was performed using a three-port technique. Pneumoperitoneum was established using a Veress needle inserted through an infraumbilical incision, maintaining an intra-abdominal pressure of 10 to 12 mmHg. A 30° 5-mm laparoscope was inserted, and two additional 5-mm ports were placed under direct vision: one in the midclavicular line and the other in the anterior axillary line, on the side of the affected kidney. After medial mobilization of the colon, the ureter and renal vessels were carefully dissected. The renal artery and vein were clipped with Hem-o-lok polymer clips and divided. The ureter was divided distally after tracing it below the pelvic brim. The kidney was mobilized, placed in an endoscopic retrieval bag, and extracted through an extended port site.

Intraoperative variables, including operative time (skin incision to closure), blood loss (estimated by suction container and swab weight), and the need to convert to open surgery, were documented. Postoperative parameters recorded included time to initiation of oral intake, requirement for parenteral analgesia, duration of hospital stay, and complications within 30 days (Clavien-Dindo classification used). Follow-up visits were scheduled at two weeks, six weeks, and three months to evaluate late complications and recovery outcomes.

Using IBM SPSS Statistics for Windows, Version 26 (Released 2018; IBM Corp., Armonk, NY, USA), descriptive statistics were calculated for all variables. Continuous variables (e.g., age, operative duration, blood loss, and duration of hospital stay) were reported as mean ± standard deviation after checking for normality of the data with the Shapiro-Wilk test. As most continuous data showed a normal distribution, independent samples t-test was used to compare means between subgroups (e.g., age groups and right vs. left nephrectomy). Categorical variables (e.g., gender, conversion to open surgery, and complications) were expressed as frequencies and percentages, and associations were analyzed using the Chi-square test. A p-value < 0.05 was considered statistically significant.

Written informed consent was obtained from the parents or legal guardians of all participants after explaining the purpose, benefits, and risks of the procedure. Patient confidentiality was maintained in accordance with institutional and ethical guidelines.

## Results

A total of 102 pediatric patients underwent laparoscopic nephrectomy for non-functioning hydronephrotic kidneys. The cohort had a median age of seven years and a male predominance (60.8%). PUJO was the most common pathology (45.1%), with the left kidney more frequently involved. The procedure was successfully completed laparoscopically in 94.1% of cases, with a low conversion rate of 5.9%. Operative time and blood loss were within acceptable limits, and intraoperative complications occurred in only 4.9% of cases - all managed without major morbidity. Postoperative recovery was favorable, with a median hospital stay of four days and minimal complications (6.9%), primarily low-grade infections and urinary issues. Hematologic and renal function parameters showed only slight perioperative changes, indicating stable physiological status (Table 1).

**Table 1:** Demographic, Surgical, Postoperative, and Laboratory Characteristics of Study Participants (n = 102) Values are expressed as n (%) for categorical variables (+), and mean ± standard deviation (#) for continuous variables unless otherwise stated. Median is specified where applicable. PUJO, Pelviureteric Junction Obstruction; VUR, Vesicoureteral Reflux

Parameter	Value
Median Age (years)	7
Gender	
- Male	62 (60.8%) ^+^
- Female	40 (39.2%) ^+^
Side of Kidney Involved	
- Right	45 (44.1%) ^+^
- Left	57 (55.9%) ^+^
Type of Pathology	
- PUJO	46 (45.1%) ^+^
- Stone Disease	39 (38.2%) ^+^
- VUR	17 (16.7%) ^+^
Laparoscopic Procedure Completed	96 (94.1%) ^+^
Conversion to Open Surgery	6 (5.9%) ^+^
Mean Operative Time (minutes)	93.5 ± 18.7 ^#^
Mean Estimated Blood Loss (mL)	72.4 ± 25.6 ^#^
Intraoperative Complications	5 (4.9%) ^+^
- Controlled Bleeding	3
- Bowel Serosal Injury	1
- Renal Vein Tear (converted)	1
Drainage Duration	
- 1 Day	47 (46.1%) ^+^
- 2 Days	46 (45.1%) ^+^
- 3 Days	9 (8.8%) ^+^
Requirement of Blood Transfusion	
- Yes	3 (2.9%) ^+^
- No	99 (97.1%) ^+^
Median Hospital Stay (days)	4
Postoperative Complications	7 (6.9%) ^+^
- Wound Infection (Grade I)	3
- Fever >48h (Grade II)	2
- Urinary Leak (Grade II)	2
Mean Preoperative Hemoglobin (g/dL)	11.6 ± 0.15 ^#^
Mean Postoperative Hemoglobin (g/dL)	11.0 ± 0.15 ^#^
Mean Preoperative Creatinine (mg/dL)	0.52 ± 0.61 ^#^
Mean Postoperative Creatinine (mg/dL)	0.48 ± 0.58 ^#^

A subgroup analysis revealed that conversion to open surgery was significantly associated with longer operative time (121.0 ± 16.9 vs. 91.7 ± 17.4 minutes, p = 0.001) and greater blood loss (115.7 ± 32.1 vs. 69.3 ± 22.5 mL, p = 0.004). Intraoperative complications were also significantly more frequent in the converted group (p < 0.001) (Table 2).

**Table 2:** Comparison Between Laparoscopic and Converted Cases

Variable	Laparoscopic (n = 96)	Converted (n = 6)	p-value
Mean Operative Time (min)	91.7 ± 17.4	121.0 ± 16.9	0.001
Estimated Blood Loss (mL)	69.3 ± 22.5	115.7 ± 32.1	0.004
Intraoperative Complications	2 (2.1%)	3 (50%)	<0.001
Mean Hospital Stay (days)	3.1 ± 1.0	5.2 ± 1.2	0.021

At two-week, six-week, and three-month follow-up visits, all patients demonstrated complete wound healing, no delayed complications such as incisional hernia or obstruction, and stable renal function.

Chi-square tests were used to determine the associations between various clinical and surgical parameters. No statistically significant association was found between gender and any of the outcome variables, suggesting that gender does not affect the risk of conversion or complications. A statistically significant association was found between the type of pathology and conversion to open surgery (p = 0.034), with PUJO cases more likely to require conversion (Table 3).

**Table 3:** Association of Demographic and Clinical Variables With Surgical Outcomes in Pediatric Laparoscopic Nephrectomy (n = 102) *p < 0.05 indicates statistical significance. PUJO, Pelviureteric Junction Obstruction; VUR, Vesicoureteral Reflux

Variable	Categories	Conversion to Open, n (%)	Intra-op Complications, n (%)	Post-op Complications, n (%)
Gender	Male (n = 62)	2 (3.2%)	4 (6.5%)	3 (4.8%)
	Female (n = 40)	2 (5.0%)	2 (5.0%)	1 (2.5%)
p-value		0.647	0.748	0.546
Side Involved	Right (n = 45)	1 (2.2%)	3 (6.6%)	2 (4.4%)
	Left (n = 57)	3 (5.3%)	3 (5.2%)	2 (3.5%)
p-value		0.412	0.782	0.832
Type of Pathology	PUJO (n = 46)	3 (6.5%)	3 (6.5%)	2 (4.3%)
	Stones (n = 39)	1 (2.6%)	2 (5.1%)	1 (2.6%)
	VUR (n = 17)	0 (0.0%)	1 (5.9%)	1 (5.9%)
p-value		0.034*	0.961	0.821

## Discussion

The median age of our patients was seven years, with the majority being male (60.8%). This demographic trend is consistent with studies by Iqbal et al. [13] and Ezomike et al. [14], who reported similar gender distributions, with 61.6% and 61.5% of patients being male, respectively. Molina et al. also reported a mean age of 46.5 months, indicating that the age at which laparoscopic nephrectomy is performed varies across institutions, often influenced by local referral patterns and surgical expertise [15].

In our cohort, the left kidney was more frequently involved (55.9%) than the right (44.1%). This mirrors the findings of Ezomike et al. [14] and Zafar et al. [4], who noted left-sided predominance (59%) [2], though studies like Iqbal et al. [13] have reported more right-sided cases (53%). These differences likely reflect random distribution rather than anatomical or pathological predisposition.

PUJO was the most common indication for nephrectomy (45.1%), followed by stone disease (38.2%) and VUR (16.7%). These findings are comparable to a large series where PUJO accounted for 47% of cases, and also agree with patterns seen in published literature, where PUJO and stones were dominant causes [16,17]. In terms of procedural safety, the postoperative outcomes in our cohort were favorable, with only seven patients (6.9%) developing complications, all of which were classified as Clavien-Dindo Grade I-II. The complications included minor wound infections, transient fever, and urinary leakage, all managed conservatively without surgical intervention. Importantly, no Grade III-V complications were observed, indicating the absence of major adverse events. This pattern aligns with previous reports [1], where the authors observed only low-grade complications following pediatric laparoscopic nephrectomy. The predominance of low-grade, self-limiting complications further reinforces the safety, minimal invasiveness, and feasibility of the laparoscopic approach in managing hydronephrotic, non-functioning kidneys in children.

We achieved a 94.1% success rate in completing the procedure laparoscopically, with a 5.9% conversion rate to open surgery. This is well within the reported range from other studies. For example, Negi et al. reported a conversion rate of 6% [5], Ekşi et al. found a rate of 10.3% [18], and Ibrahium et al. observed conversion in 10.3% of cases [19]. In contrast, Zhou et al. reported no conversions [20], which may reflect increased surgeon experience or different case complexity. Our higher conversion rate in PUJO cases (statistically significant, p = 0.034) may be explained by the distorted anatomy and adhesions commonly associated with longstanding obstruction, consistent with challenges noted by Mishra et al. [16].

The mean operative time in our study was 93.5 ± 18.7 minutes, which is lower than that reported by Iqbal et al. (173 minutes) [13] and also lower than in Menon et al. (138 minutes) [21], suggesting improved efficiency, possibly due to a single-surgeon protocol and case selection. Studies with higher mean durations often reflect early learning curves or more complex cases [21-23].

Intraoperative complications were encountered in 4.9% of patients, including controlled bleeding, one serosal bowel injury, and a renal vein tear (leading to conversion). This aligns with the 8.3% complication rate reported by Mehmood et al. [24] and remains below the 18% complication rate reported by Tsai et al. [25]. Careful port placement, controlled insufflation, and clear visibility are vital, as emphasized by Esposito et al. [26] and Menon et al. [21], in reducing such events.

The median hospital stay was four days, similar to reports by Iqbal et al., who reported 3.4 days, and Zafar et al., who reported four days [4]. In our study, drains were typically removed by day 2, and patients were discharged after stable vitals and adequate oral intake were ensured. Postoperative complications occurred in only 6.9% of patients, all of which were low-grade (Clavien-Dindo I/II). The most common issues were minor wound infections and fever. These findings are consistent with other studies; for example, Iqbal et al. reported complications in 7.7% [13]. Importantly, no patient in our study required reoperation, and all patients had uneventful recovery with normal follow-up findings up to three months.

Laboratory parameters showed only minor changes, with a drop in hemoglobin from 11.6 to 11.0 g/dL and a slight reduction in serum creatinine. These stable biochemical markers support the safety of laparoscopic nephrectomy in preserving overall patient health, as corroborated by Alsunbul et al. [27].

Subgroup analysis revealed a statistically significant association between conversion to open surgery and longer operative times, increased blood loss, and more frequent intraoperative complications (p < 0.05), reinforcing findings from published literature, where early institutional experience and complex anatomy contributed to higher conversion rates [17,18]. Further analysis in our cohort showed that PUJO was the most frequent indication among patients who required conversion, showing a statistically significant relationship (p = 0.034). The likely explanation is the presence of dense perihilar adhesions and distorted anatomical planes secondary to long-standing obstruction, which limit safe laparoscopic dissection. Similar findings have been described by other authors, who reported PUJO and extensive scarring as key predictors of conversion in pediatric laparoscopic nephrectomy [14,16]. Notably, gender and side of the kidney showed no significant association with conversion or complications, consistent with previous pediatric series [1,14]. These observations suggest that careful preoperative imaging assessment and surgical planning are essential for anticipating difficult cases and minimizing conversion rates.

The use of a single experienced surgeon ensured procedural uniformity and consistency in operative technique, which strengthens the internal validity of our results. However, we recognize this as a methodological limitation that may influence external generalizability. Future multi-surgeon or multi-center studies are recommended to further validate these outcomes and minimize potential operator-related bias.

## Conclusions

Our findings confirm that transperitoneal laparoscopic nephrectomy is a feasible, safe, and effective approach in children with hydronephrotic, non-functioning kidneys. Despite inherent challenges such as distorted anatomy and inflammation, outcomes remain favorable with appropriate surgical planning. These results are consistent with those reported internationally, affirming that - with adequate experience and case selection - laparoscopic nephrectomy can be confidently adopted in pediatric urological practice.
